# Genetic Validation of Psoriasis Phenotyping in UK Biobank Supports the Utility of Self-Reported Data and Composite Definitions for Large Genetic and Epidemiological Studies

**DOI:** 10.1016/j.jid.2023.02.010

**Published:** 2023-08

**Authors:** Jake R. Saklatvala, Ken B. Hanscombe, Satveer K. Mahil, Lam C. Tsoi, James T. Elder, Jonathan N. Barker, Michael A. Simpson, Catherine H. Smith, Nick Dand

**Affiliations:** 1 Department of Medical and Molecular Genetics, King’s College London, London, United Kingdom; 2 Social, Genetic and Developmental Psychiatry Centre, King's College London, London, United Kingdom; 3 St John's Institute of Dermatology, Guy's and St Thomas' NHS Foundation Trust, King's College London, London, United Kingdom; 4 Department of Dermatology, University of Michigan Medical School, Ann Arbor, Michigan, USA; 5 Department of Computational Medicine and Bioinformatics, University of Michigan Medical School, Ann Arbor, Michigan, USA; 6 Department of Biostatistics, School of Public Health, University of Michigan, Ann Arbor, Michigan, USA; 7 Ann Arbor Veterans Affairs Hospital, Ann Arbor, Michigan, USA

To the Editor

In dermatology and elsewhere, GWAS meta-analyses now routinely include data from large-scale population-based biobanks (Zhou et al., 2022). Many examples (Boutin et al., 2020; Han et al., 2020; Mitchell et al., 2022; Tachmazidou et al., 2019) have used data from UK Biobank, a study of >500,000 participants aged 40–70 years with self-reported and electronic health record–derived clinical diagnoses (Bycroft et al., 2018). However, correct interpretation of genetic or epidemiological associations identified in biobank data should acknowledge that cases selected via study-specific self-report and electronic health record procedures may be subject to misclassification or a different disease phenotype on average than those ascertained in a specialist clinical setting and typically used in molecular studies of disease processes (Cai et al., 2020).

We focus on chronic plaque psoriasis, reporting a framework that uses genetic effect size estimates to evaluate the consistency between candidate biobank phenotypes and psoriasis diagnosed by a specialist physician. Specifically, we assess the degree to which candidate biobank definitions capture nonpsoriasis cases—or (presumably milder) psoriasis cases with lower genetic liability than typical specialist-ascertained cases—by regressing estimated genetic effect sizes at established psoriasis susceptibility loci against reference values obtained from a previous GWAS of psoriasis case cohorts in which recruitment was based on in-person specialist diagnosis (Tsoi et al., 2017) (Figure 1). Our inverse variance–weighted regression slope estimates a lower bound for the positive predictive value (minPPV) for true psoriasis cases among participants selected by the candidate biobank definition (full details are provided in Supplementary Materials and Methods). We validate our approach on dermatologist-derived case-control psoriasis GWASs and simulated case-control cohorts with known misclassification rate (Supplementary Materials and Methods, Supplementary Figure S1, and Supplementary Table S4).

**Figure 1:**
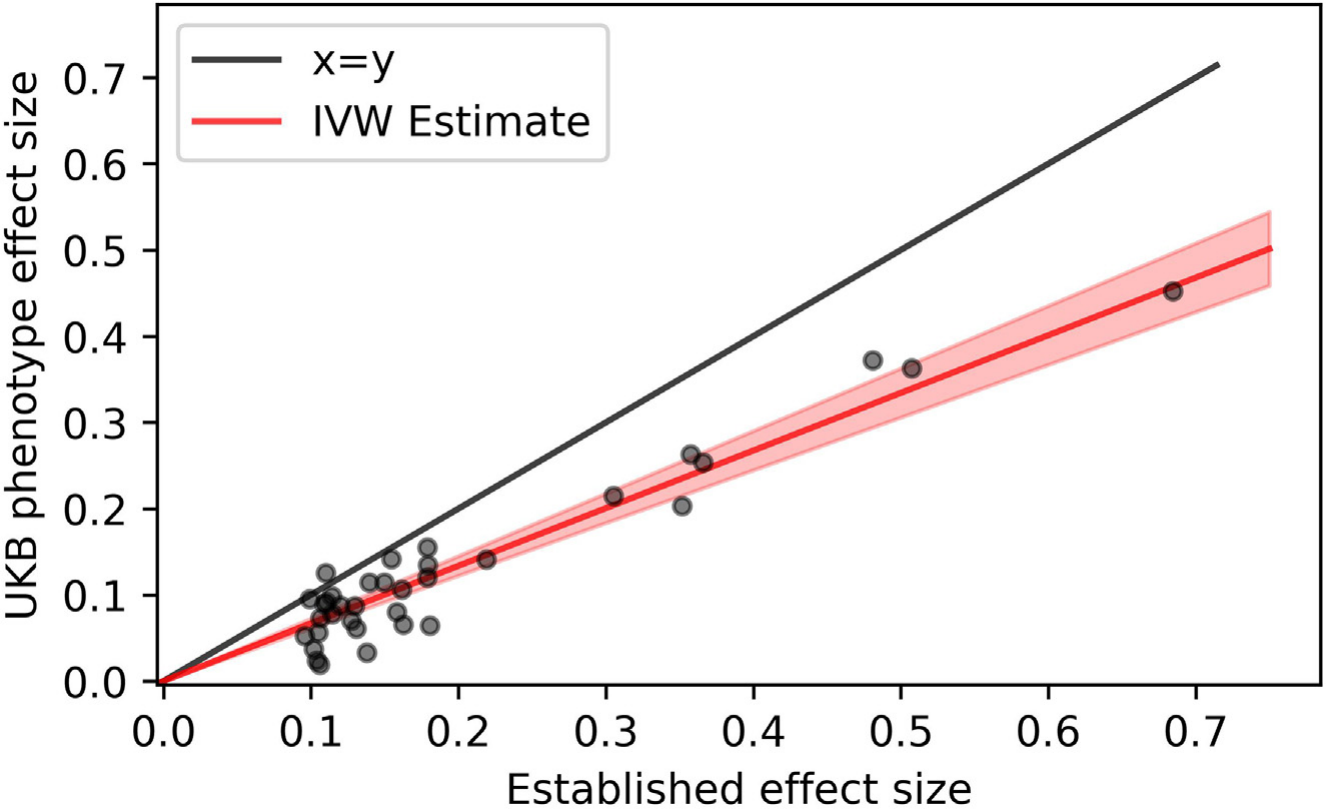
**Illustration of regression approach used to assess candidate phenotype diagnostic minPPV (IVW estimate).** UK Biobank self-reported psoriasis effect size (y-axis) plot against Tsoi et al. specialist-diagnosed cohort effect size (x-axis) for 35 independent psoriasis risk SNVs. Red line is IVW estimated slope, with the shaded area indicating a 95% CI. CI, confidence interval; IVW, inverse variance–weighted; minPPV, lower bound of positive predictive value for psoriasis phenotype (i.e., IVW regression slope).

We applied our method to UK Biobank (unrelated White British participants after quality control; N = 336,733), in which psoriasis cases can be defined using a single data source (self-reporting, linked general practitioner [GP] diagnoses or Hospital Episode Statistics; Table 1, Supplementary Table S1), or combinations thereof. Among single-source candidate psoriasis definitions, self-reported psoriasis (N_SRP_ = 4,244) was most concordant with specialist-diagnosed psoriasis (minPPV_SRP_ = 66.9%, 95% confidence interval [CI]: 61.2–72.6%), even more so with a self-reported psoriasis-relevant medication (N = 1,927; minPPV = 73.9%, 95% CI: 65.2–82.6%). Psoriasis definitions from Hospital Episode Statistics (HES) identified fewer psoriasis cases (N_HESany_ = 1,726) and were less concordant (minPPV_HESany_ = 57.9%, 95% CI: 48.9–66.8%). GP-based psoriasis definitions were least concordant with specialist diagnosis (N_GP_ = 5,768; minPPV_GP_ = 46.4%, 95% CI: 40.5–52.3%), albeit improving when multiple GP diagnoses were required (N_GP2_ = 2,422; minPPV_GP2_ = 58.6%, 95% CI: 50.3–66.9%).

**Table 1:** List of Selected Candidate UK Biobank Psoriasis Phenotypes, with Abbreviations, Case Numbers before and after Genotyping QC, IVW Estimate, and Power to Detect a Common (MAF = 30%) Risk Factor of Weak Effect (OR = 1.1)

Abbreviation	Phenotype Description	Number of Psoriasis Cases (All)	Number of Psoriasis Cases (Genotyped, White British Unrelated)	IVW Regression Slope (∼minPPV) Mean (95% CI) (vs Selected Controls, n = 141,279)	Power to Detect Common Weak Effect (vs Selected Controls)
**Single data source**					
SRP	Self-reported psoriasis	6,110	4,244	0.669 (0.612–0.726)	0.478
SRPM	Self-reported psoriasis and medication relevant to psoriasis	2,750	1,927	0.739 (0.652–0.826)	0.296
HESmain	Psoriasis as main diagnosis in linked HES	449	289	0.605 (0.422–0.788)	0.077
HESsec	Psoriasis as secondary diagnosis in linked HES	2,300	1,532	0.587 (0.491–0.683)	0.175
HESany	Psoriasis as main or secondary diagnosis in linked HES	2,593	1,726	0.579 (0.489–0.668)	0.178
GPraw	Psoriasis diagnosis in linked GP data, using read codes corresponding to ICD-10 psoriasis codes in UK Biobank mapping file	11,560	7,956	0.324 (0.279–0.370)	0.243
GP	Psoriasis diagnosis in linked GP data, using curated list of read codes	8,444	5,768	0.464 (0.405–0.523)	0.340
GP2	Two or more psoriasis diagnoses in GP data using curated read codes	3,472	2,422	0.586 (0.503–0.669)	0.242
GP3	Three or more psoriasis diagnoses in GP data using curated read codes	1,984	1,389	0.614 (0.515–0.714)	0.172
**Combined data sources**					
1-SRP-HESany	Any one of SRP or HESany	7,568	5,194	0.624 (0.570–0.677)	0.499
1-SRP-GP	Any one of SRP or GP	12,616	8,647	0.517 (0.471–0.563)	0.543
1-SRP-GP2	Any one of SRP or GP2	8,320	5,786	0.615 (0.559–0.670)	0.538
1-SRP-HESany-GP	Any one of SRP, HESany or GP	13,666	9,316	0.508 (0.463–0.553)	0.561
1-SRP-HESany-GP2	Any one of SRP, HESany or GP2	9,546	6,574	0.585 (0.535–0.636)	0.542
2-SRP-HESany-GP	Any two of SRP, HESany or GP	3,056	2,122	0.721 (0.638–0.805)	0.303
All-SRP-HESany-GP	All three of SRP, HESany or GP	425	300	0.818 (0.616–1.020)	0.105
2-SRP-SRM-HESany-GP	Any two of SRP, SRM, HESany or GP	5,080	3,499	0.696 (0.628–0.763)	0.443
2-SRP-SRM-HESany-GP2	Any two of SRP, SRM, HESany or GP2	4,291	2,965	0.726 (0.660–0.792)	0.416
3-SRP-SRM-HESany-GP	Any three of SRP, SRM, HESany or GP	1,599	1,122	0.771 (0.675–0.866)	0.216
All-SRP-SRM-HESany-GP	All four of SRP, SRM, HESany or GP	262	185	0.870 (0.637–1.104)	0.084
**Phenotypes incorporating PsA codes**					
SRP+PsA	Self-reported psoriasis or psoriatic arthritis	6,636	4,603	0.664 (0.606–0.721)	0.503
SRPM+PsA	Self-reported psoriasis or PsA, and psoriasis-relevant medication	3,013	2,107	0.747 (0.661–0.832)	0.330
HESany+PsA	Psoriasis or PsA as main or secondary diagnosis in linked HES	3,388	2,272	0.616 (0.530–0.703)	0.252
GP+PsA	Psoriasis or PsA diagnosis in linked GP data using curated read codes	8,808	6,024	0.457 (0.398–0.517)	0.348
1-SRP-HESany-GP+PsA	Any one of SRP+PsA, HESany+PsA or GP+PsA	14,475	9,864	0.510 (0.464–0.555)	0.582
2-SRP-HESany-GP+PsA	Any two of SRP+PsA, HESany+PsA or GP+PsA	3,719	2,579	0.713 (0.629–0.797)	0.348
2-SRP-SRM-HESany-GP+PsA	Any two of SRP+PsA, SRM+PsA, HESany+PsA or GP+PsA	5,692	3,917	0.688 (0.620–0.756)	0.468

Abbreviations: CI, confidence interval; GP, general practitioner; HES, Hospital Episode Statistics; IVW, inverse variance-weighted; MAF, minor allele frequency; minPPV, lower bound of positive predictive value for psoriasis phenotype (i.e., IVW regression slope); OR, odds ratio; PsA, psoriatic arthritis; QC, quality control; SRP, self-reported psoriasis; SRPM, self-reported psoriasis-relevant medication.

We recognize that the large sample sizes afforded by biobank studies may offset limitations in phenotype stringency when considering statistical power to detect novel genetic and epidemiological associations (Supplementary Figure S2). We therefore estimated the power to detect an association with a novel psoriasis risk factor (population frequency 0.3, odds ratio 1.1; Table 1) (details and results for other scenarios are presented in Supplementary Materials and Methods and Supplementary Figure S3). Among single-source candidate definitions in UK Biobank, self-reported psoriasis demonstrated the highest power for discovery (power_SRP_ = 47.8%), substantially higher than the larger but less concordant GP-based definition (power_GP_ = 34.0%).

We then considered composite psoriasis definitions based on multiple data sources. Requiring a single coding from any source conferred limited agreement with specialist-defined psoriasis (minPPV_1-SRP-HESany-GP_ = 50.8%, 95% CI: 46.3–55.3%) but large case numbers such that statistical power for discovery exceeded all other definitions (N_1-SRP-HESany-GP_ = 9,316; power_1-SRP-HESany-GP_ = 56.1%). Requiring two independent corroborative codings improved concordance with specialist-defined psoriasis to ∼70% (minPPV_2-SRP-HESany-GP_ = 72.2%, 95% CI: 63.8–80.5%; minPPV_2-SRP-SRM-HESany-GP_ = 69.6%, 95% CI: 62.8–76.3%) although power (power_2-SRP-HESany-GP_ = 30.3%; power_2-SRP-SRM-HESany-GP_ = 44.3%) remained lower than the top-performing single-source definition (power_SRP_ = 47.8%). UK Biobank participants with psoriasis codings across all data sources demonstrated high concordance (minPPV_All-SRP-HESany-GP_ = 81.8%, 95% CI: 61.6–102.0%; minPPV_All-SRP-SRM-HESany-GP_ = 87.0%, 95% CI: 63.7–110.4%) with CIs crossing 100%. This is consistent with our positive control GWASs, which had slope estimates between 0.9 and 1.1 with CIs crossing 1 (Supplementary Table S4), the smallest cohort (n = 464 cases) being the only exception.

Our estimated minPPV of self-reported psoriasis in the UK Biobank (67%) is much higher than previous self-reported psoriasis in 23andMe (36%) (Tsoi et al., 2017). This may be due to ascertainment differences: rather than an online questionnaire, UK Biobank participants are interviewed by a trained research nurse and are required to have seen a doctor for each reported condition (UK Biobank, 2012). Primary care and hospital data may have lower estimated minPPVs than self-reporting owing to misclassification because of the difficulty in the nonspecialist differential diagnosis of psoriasis from other common lesional skin diseases. Alternatively, patients diagnosed through primary care or hospital episodes (in which most recorded diagnoses are secondary) may have milder psoriasis on average, with consequently reduced genetic liability, than those included in dermatologist-diagnosed psoriasis GWASs; previous work showed that 90% of psoriasis primary care diagnoses were subsequently confirmed by GP reviewers (Seminara et al., 2011). The relatively low regression slope estimates for UK Biobank psoriasis indicators may represent not only case misclassification but also a lower genetic liability for psoriasis among patients with mild disease than those with severe disease. We recognize that without a formal validation exercise, methods such as those presented here are unable to distinguish between these scenarios. However, when considering that most molecular research into psoriasis biology is conducted in patients with moderate-severe psoriasis, our inverse variance–weighted slope estimates remain valuable as a measure of aggregate genetic risk among cases equivalent to a positive predictive value for dermatologist-ascertained psoriasis.

The optimal psoriasis definition for future genetic and epidemiological investigations will depend on the specific research aims. In UK Biobank, we recommend that discovery research, with statistical power a priority, defines cases using any self-reported or electronic health record psoriasis coding (and our maximum statistical power of 58% should be interpreted in the context of contributing to larger meta-analyses); studies requiring accurate effect size estimates and high concordance with dermatologist-diagnosed psoriasis are encouraged to use two or more data sources. We also recommend the inclusion of psoriatic arthritis diagnostic codes for the beneficial effect on sample size with minimal drop-off in concordance (Supplementary Table S2). It remains unclear whether concordance is unaffected because of the psoriatic arthritis–only participants having cutaneous psoriasis not coded in UK Biobank, or because psoriatic arthritis shares genetic risk loci with cutaneous psoriasis. In UK Biobank, a definition requiring only self-reporting of psoriasis balances both high diagnostic validity and statistical power; generalization of this finding to other datasets may depend on the ascertainment method. To facilitate such assessments, we have demonstrated here an approach to assess the composition of psoriasis diagnoses when assembling future cohorts from large electronic health record/questionnaire-based biobank studies.

### Data availability statement

The UK Biobank resource is available to bona fide researchers for health-related research in the public interest (https://www.ukbiobank.ac.uk/enable-your-research).

Biomarkers of Systemic Treatment Outcomes in Psoriasis (BSTOP) data are available for approved research use by making an application to the BSTOP Data Access Committee (https://www.kcl.ac.uk/lsm/research/divisions/gmm/departments/dermatology/research/stru/groups/bstop/documents).

## ORCIDs

Jake R. Saklatvala: http://orcid.org/0000-0003-0836-4928

Ken B. Hanscombe: http://orcid.org/0000-0002-3715-6805

Satveer K. Mahil: http://orcid.org/0000-0003-4692-3794

Lam C. Tsoi: http://orcid.org/0000-0003-1627-5722

James T. Elder: http://orcid.org/0000-0003-4215-3294

Jonathan N. Barker: http://orcid.org/0000-0002-9030-183X

Michael A. Simpson: http://orcid.org/0000-0002-8539-8753

Catherine H. Smith: http://orcid.org/0000-0001-9918-1144

Nick Dand: http://orcid.org/0000-0002-1805-6278

## Conflict of Interest

SKM reports departmental income from AbbVie, Almirall, Eli Lilly, Novartis, Sanofi, and UCB, outside the submitted work. CHS is principal investigator on MRC (PSORT) and EC-funded consortia with multiple industry partners (see PSORT.org.uk, BIOMAP-IMI.eu, and HIPPOCRATES-IMI.eu for up-to-date listings of contributory partners), and is a co-supervisor of PhD studentships through MRC/industry collaboration (Boehringer Ingelheim GmbH). The remaining authors state no conflict of interest.

## Figures and Tables

**Supplementary Figure S1:**
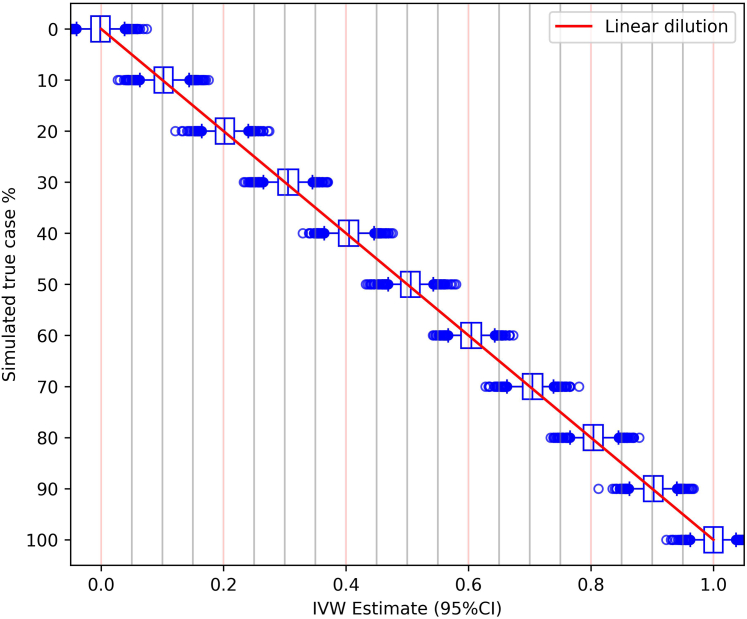
**Simulations to characterize the impact of psoriasis case dilution on observed effect sizes.** IVW estimates for each simulated PPV (1,000 simulations at each case dilution). Boxplots indicate the median and lower and upper quartiles of the simulations, with whiskers indicating 5th and 95th percentile and outliers shown as blue circles. Red line indicates theoretical linear dilution where IVW estimate corresponds to PPV. IVW, inverse variance–weighted; PPV, positive predictive value.

**Supplementary Figure S2:**
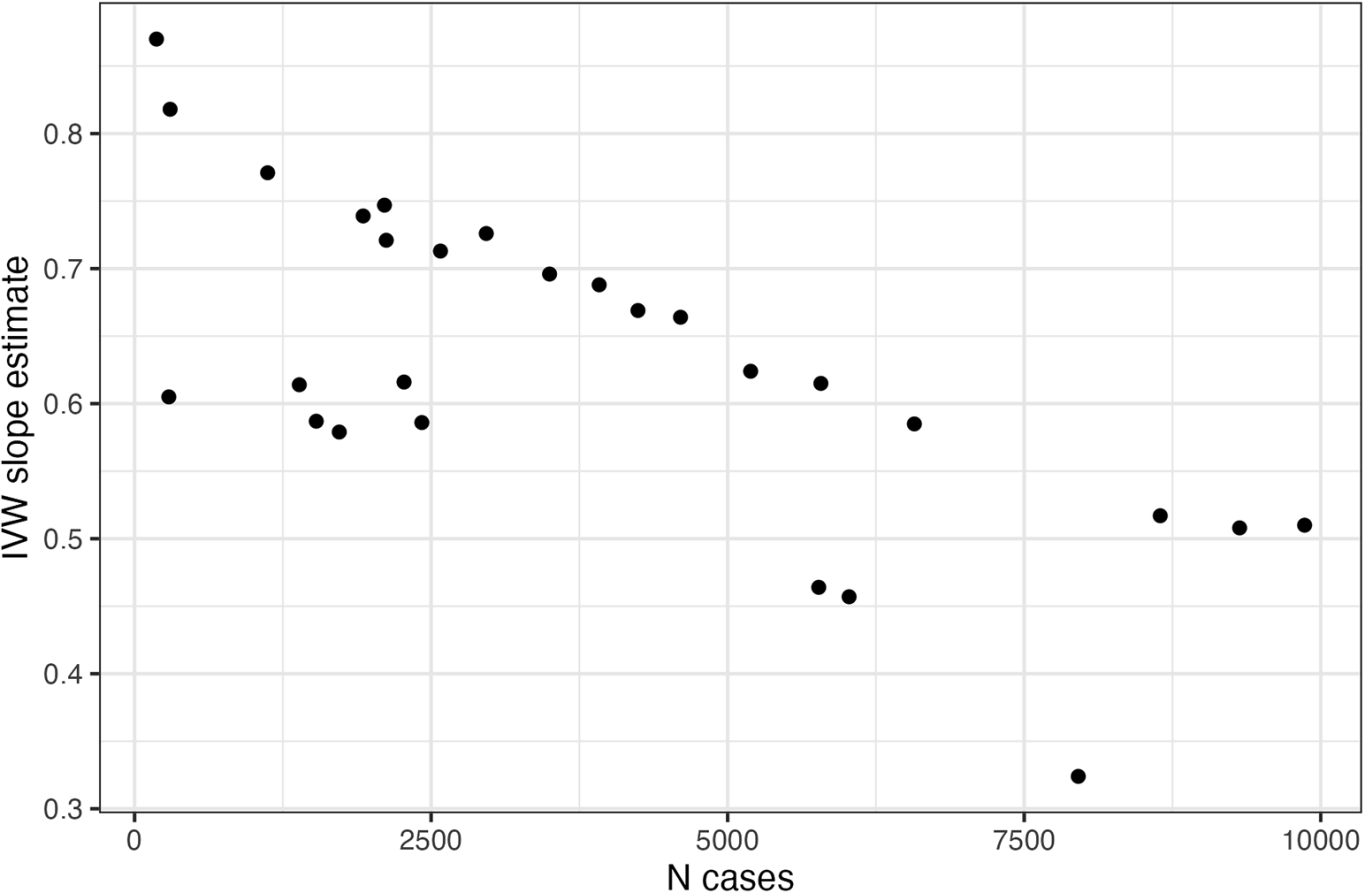
**IVW slope estimate by number of cases across all candidate UK Biobank phenotypes.** IVW slope estimate plot against the number of cases for each of the UK Biobank psoriasis definitions in Supplementary Table S2. IVW, inverse variance–weighted.

**Supplementary Figure S3:**
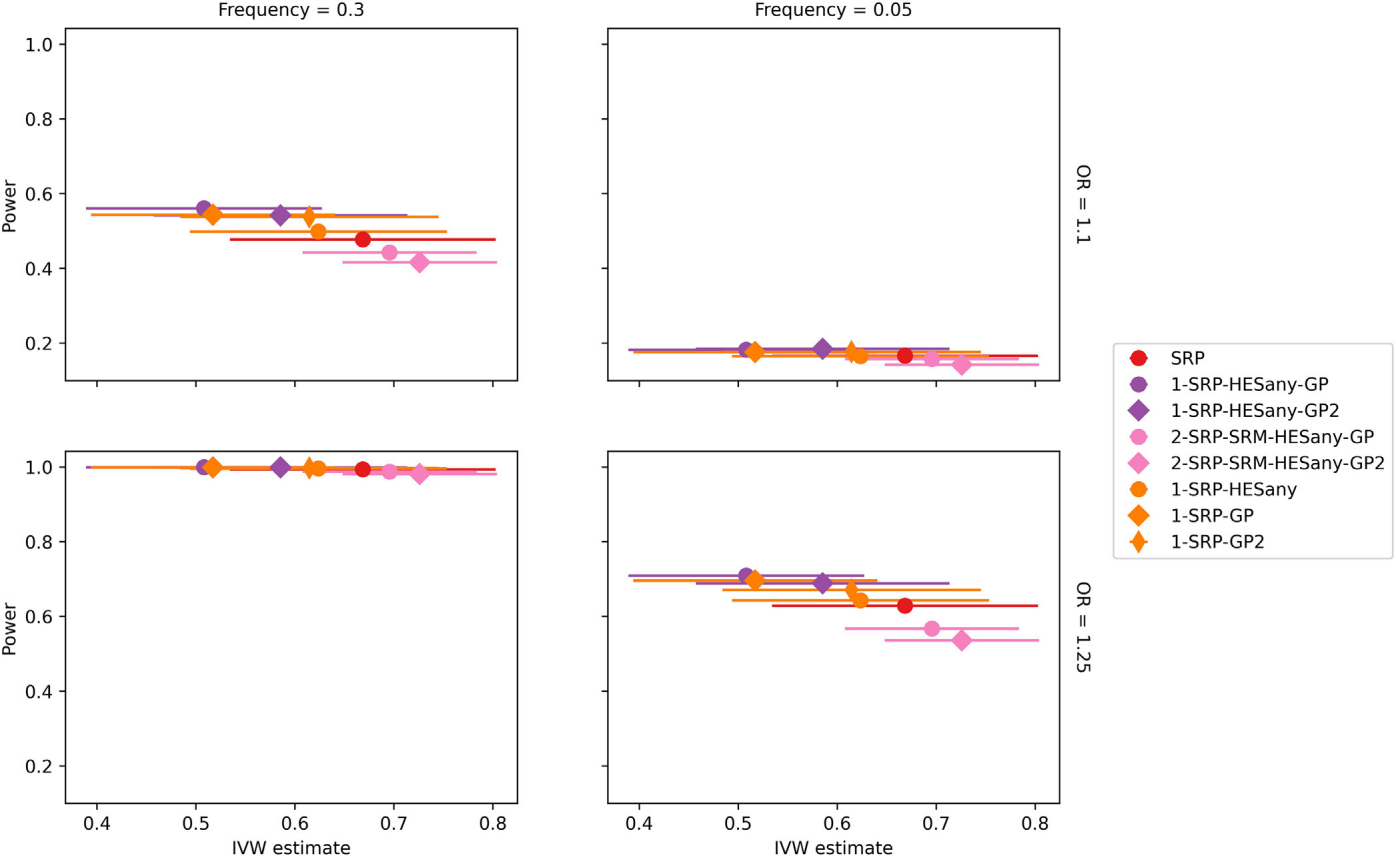
**Power to detect novel psoriasis-associated risk factors for top-performing candidate psoriasis definitions.** Comparison of phenotype IVW estimate (minPPV) and statistical power to detect novel psoriasis-associated risk factors under various scenarios for selected candidate psoriasis definitions that score highly on both measures. Y-axis: estimated statistical power; x-axis: IVW estimate of candidate psoriasis definition; left column: common risk factor (frequency 0.3); right column: rare risk factor (frequency 0.05); top row: weak risk factor (odds ratio 1.1); bottom row: strong risk factor (odds ratio 1.25). Candidate phenotype abbreviations are described in Table 1. IVW, inverse variance–weighted; minPPV, lower bound for the positive predictive value.

**Supplementary Figure S4:**
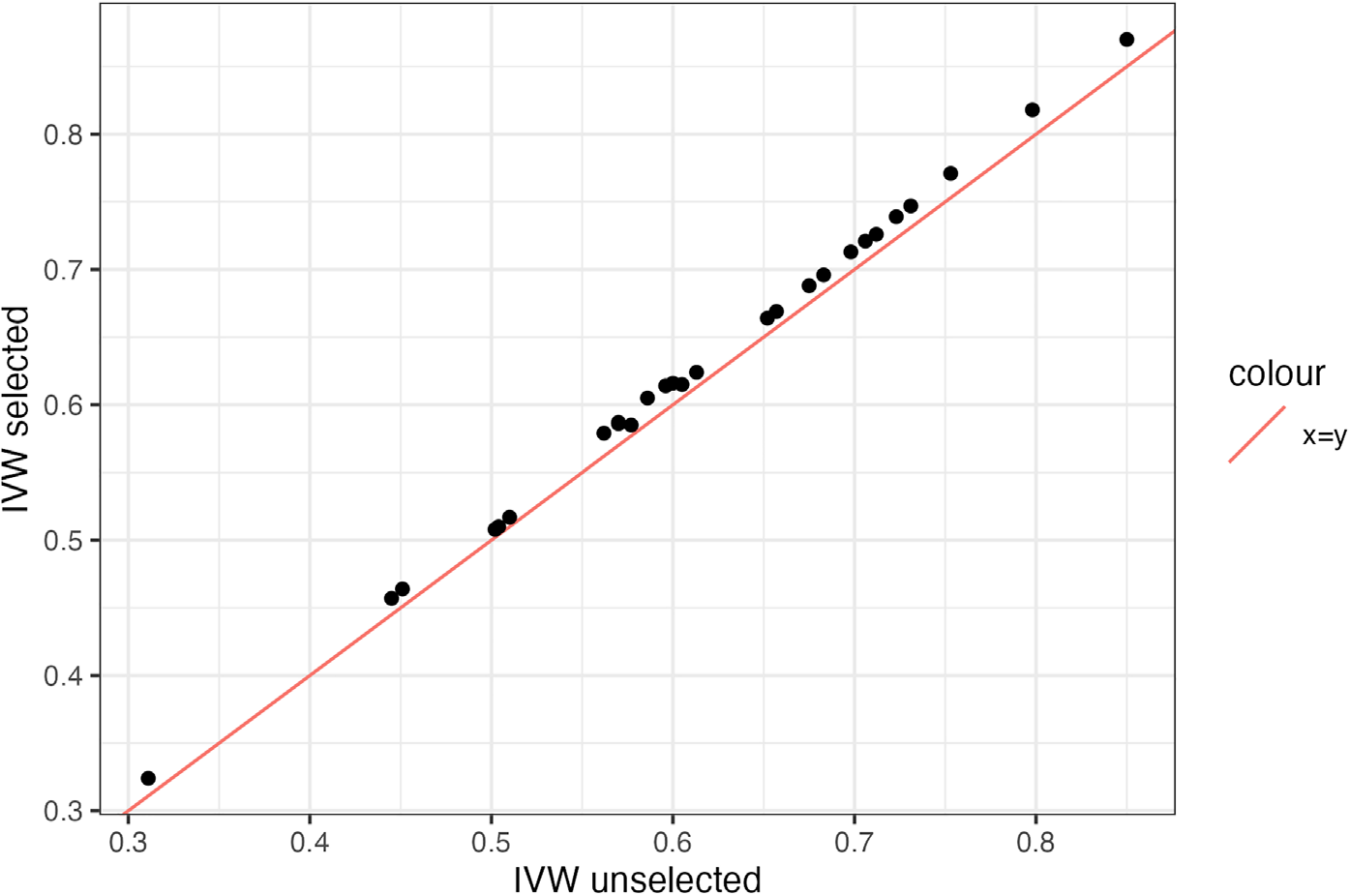
**Comparison of candidate psoriasis definition IVW using selected and unselected controls.** IVW slope estimates for UK Biobank psoriasis definitions with selected controls (Supplementary Table S2) plot against IVW slope estimate for equivalent UK Biobank psoriasis definitions with unselected controls (Supplementary Table S3). Red line (x = y) represents equality. IVW, inverse variance–weighted.

**Supplementary Figure S5:**
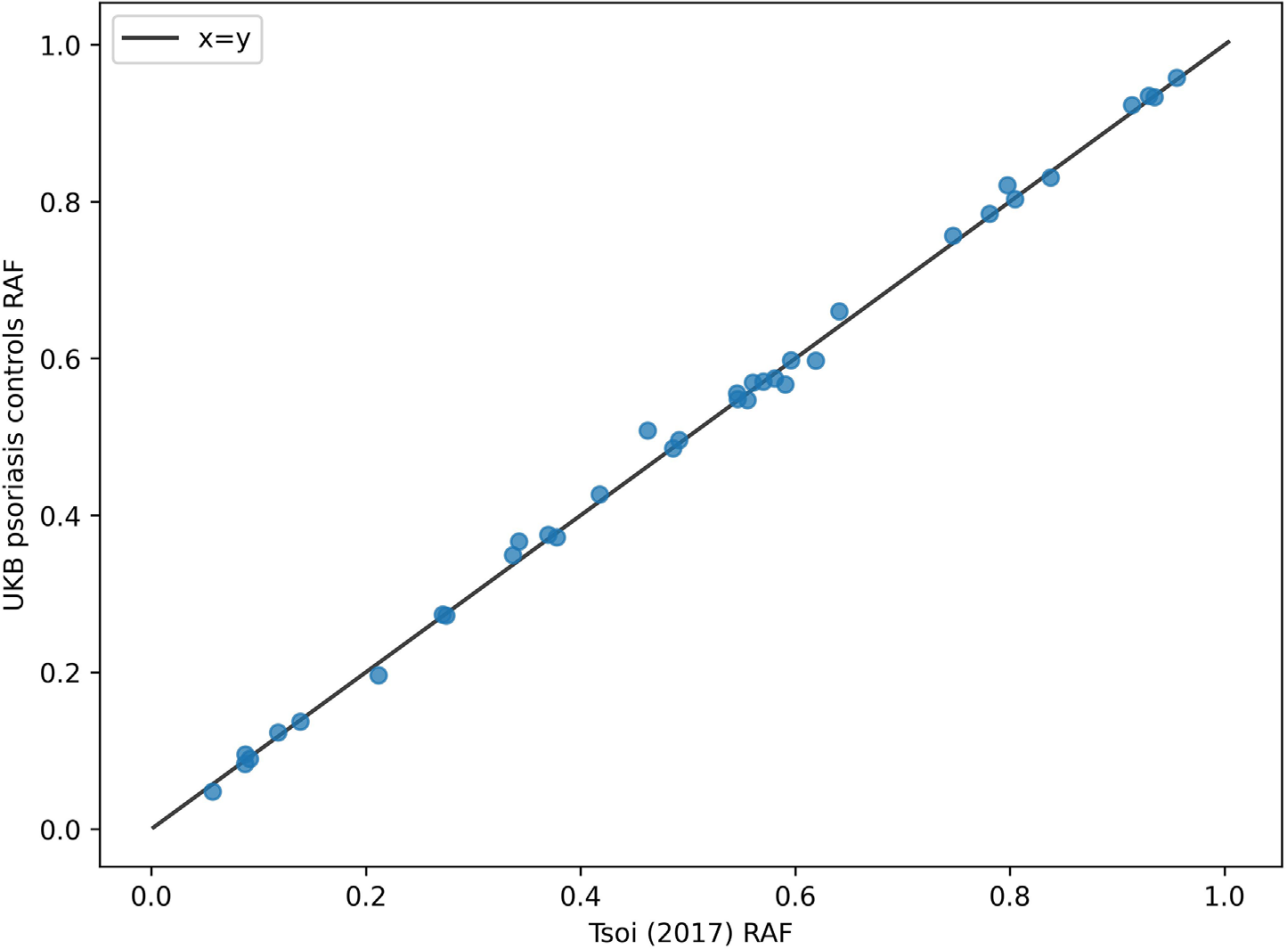
**Comparison of risk allele frequencies between UK Biobank psoriasis controls (N = 141,279) and Tsoi et al. (2017) meta-analysis controls (N = 20,154).** Risk allele frequency in UK Biobank psoriasis controls (y-axis) plotted against risk allele frequency in Tsoi et al. (2017) psoriasis controls, for SNVs in Supplementary Table S5 and HLA locus lead SNV (rs12189871).

**Supplementary Table S1:** UK Biobank Data Fields and Codes to Define Single-Source Candidate Psoriasis Phenotypes

Abbreviation	Phenotype Description	UK Biobank Data Fields	Codes Used
SRP	Self-reported psoriasis	20002	1453 – Psoriasis
SRPM	Self-reported psoriasis and medication relevant to (but not necessarily specific for) psoriasis	20003	1140854254 | 1140854432 | 1140859220 | 1140864184 | 1140864346 | 1140864820 | 1140865734 | 1140868364 | 1140869848 | 1140870980 | 1140874336 | 1140874790 | 1140874896 | 1140874930 | 1140875538 | 1140876312 | 1140876314 | 1140876404 | 1140876424 | 1140876430 | 1140878226 | 1140878244 | 1140878246 | 1140878248 | 1140878252 | 1140878258 | 1140878266 | 1140878286 | 1140878288 | 1140878304 | 1140878328 | 1140878346 | 1140878348 | 1140878378 | 1140878412 | 1140878416 | 1140878418 | 1140878426 | 1140878442 | 1140878444 | 1140878446 | 1140878448 | 1140878450 | 1140878488 | 1140878498 | 1140878504 | 1140878506 | 1140878508 | 1140878510 | 1140878512 | 1140878514 | 1140879902 | 1140879906 | 1140879926 | 1140879928 | 1140879930 | 1140879934 | 1140879938 | 1140879940 | 1140879942 | 1140880014 | 1140880086 | 1140880090 | 1140880092 | 1140880102 | 1140880256 | 1140880264 | 1140880272 | 1140880288 | 1140880306 | 1140880460 | 1140882542 | 1140882544 | 1140882618 | 1140882622 | 1140882630 | 1140882694 | 1140882708 | 1140882756 | 1140882776 | 1140882778 | 1140882782 | 1140882794 | 1140882800 | 1140882806 | 1140882818 | 1140882820 | 1140882852 | 1140882904 | 1140882906 | 1140882908 | 1140882910 | 1140882918 | 1140882920 | 1140883828 | 1140884640 | 1140884646 | 1140884654 | 1140884696 | 1140884700 | 1140884704 | 1140888074 | 1140888092 | 1140888142 | 1140888172 | 1140888176 | 1140888178 | 1140888184 | 1140888628 | 1140888630 | 1140888644 | 1140909786 | 1140909844 | 1140910036 | 1140910058 | 1140910368 | 1140910382 | 1140910416 | 1140910424 | 1140910522 | 1140910884 | 1140913292 | 1140916270 | 1140916274 | 1140916276 | 1140916948 | 1140916950 | 1140921024 | 1140923728 | 1140923732 | 1140925978 | 1140926988 | 1140927320 | 1140927730 | 1141145710 | 1141146422 | 1141146426 | 1141150430 | 1141151026 | 1141151848 | 1141152236 | 1141152242 | 1141157294 | 1141157402 | 1141157414 | 1141162940 | 1141164652 | 1141164836 | 1141167140 | 1141167744 | 1141168374 | 1141170768 | 1141170844 | 1141173346 | 1141173872 | 1141174032 | 1141174388 | 1141179842 | 1141179846 | 1141179982 | 1141179992 | 1141180200 | 1141185108 | 1141185986 | 1141187336 | 1141188588 | 1141188594 | 1141192638 | 1141195836 | 1141195844 | 1141200446 | 1141200878 | 1141200880 | 1141201130 | 2038459888
HESmain	Psoriasis as main diagnosis in linked HES	41202	L400 – Psoriasis vulgaris L401 – Generalized pustular psoriasis L402 – Acrodermatitis continua L403 – Pustulosis palmaris et plantaris L404 – Guttate psoriasis L408 – Other psoriasis L409 – Psoriasis, unspecified
HESsec	Psoriasis as secondary diagnosis in linked HES	41204	L400 – Psoriasis vulgaris L401 – Generalized pustular psoriasis L402 – Acrodermatitis continua L403 – Pustulosis palmaris et plantaris L404 – Guttate psoriasis L408 – Other psoriasis L409 – Psoriasis, unspecified
HESany	Psoriasis as main or secondary diagnosis in linked HES	41202 41204	L400 – Psoriasis vulgaris L401 – Generalized pustular psoriasis L402 – Acrodermatitis continua L403 – Pustulosis palmaris et plantaris L404 – Guttate psoriasis L408 – Other psoriasis L409 – Psoriasis, unspecified
GPraw	Psoriasis diagnosis in linked GP data, using read codes corresponding to ICD-10 psoriasis codes in UK Biobank mapping file		**V2** M07y0 | M143. | M16.. | M161. | M1610 | M1611 | M1612 | M1613 | M1614 | M1615 | M1616 | M1617 | M1618 | M1619 | M161A | M161B | M161C | M161D | M161E | M161F | M161G | M161H | M161J | M161z | M166. | M16y. | M16y0 | M16z. | Myu3. | Myu30 **CTV3** M05.. | M07y0 | M161. | M1610 | M1611 | M1612 | M1613 | M1614 | M1615 | M1616 | M1617 | M1618 | M1619 | M161A | M161B | M161C | M161D | M161E | M161F | M161G | M161z | Myu30 | X506a | X506b | X506c | X506d | X506e | X506f | X506g | X506h | X506i | X506k | X506l | X506m | X506n | X506o | X506p | X506q | X506r | X506s | X506t | X506T | X506u | X506v | X506Y | X506Z | XaNXa | XaNXt | XaYOv | XaYOx
GP (and GP2, GP3)	Psoriasis diagnosis in linked GP data, using curated list of read codes (Seminara et al., 2011)		**V2** 14F2. | M16.. | M161. | M1610 | M1611 | M1612 | M1613 | M1614 | M1615 | M1616 | M1617 | M1618 | M1619 | M161A | M161B | M161C | M161D | M161E | M161F | M161H | M161z | M16y. | M16y0 | M16z. | Myu30 **CTV3** 14F2. | M16.. | M161. | M1610 | M1611 | M1612 | M1613 | M1614 | M1615 | M1616 | M1617 | M1618 | M1619 | M161A | M161B | M161C | M161D | M161E | M161F | M161z | M16y. | M16z. | Myu30 | X506b | X506i | XaNXa | XaNXt | XaYOx
SRP+PsA	Self-reported psoriasis or PsA	20002	1453 – Psoriasis 1477 – Psoriatic arthropathy
HESany+PsA	Psoriasis or PsA as main or secondary diagnosis in linked HES	41202 41204	L400 – Psoriasis vulgaris L401 – Generalized pustular psoriasis L402 – Acrodermatitis continua L403 – Pustulosis palmaris et plantaris L404 – Guttate psoriasis L408 – Other psoriasis L409 – Psoriasis, unspecified L405 – Arthropathic psoriasis
GP+PsA	Psoriasis or PsA diagnosis in linked GP data using curated read codes (Ogdie et al., 2013; Seminara et al., 2011)		**V2** 14F2. | M16.. | M161. | M1610 | M1611 | M1612 | M1613 | M1614 | M1615 | M1616 | M1617 | M1618 | M1619 | M161A | M161B | M161C | M161D | M161E | M161F | M161H | M161z | M16y. | M16y0 | M16z. | Myu30 | M160. | M1600 | M1601 | M1602 | M160z | N0452 **CTV3** 14F2. | M16.. | M161. | M1610 | M1611 | M1612 | M1613 | M1614 | M1615 | M1616 | M1617 | M1618 | M1619 | M161A | M161B | M161C | M161D | M161E | M161F | M161z | M16y. | M16z. | Myu30 | X506b | X506i | XaNXa | XaNXt | XaYOx | M160. | M1600 | M1601 | M1602 | M160z | N0452 | X701u | X701v | X701w | X7026 | X7027 | X7028

Abbreviations: GP, general practitioner; HES, Hospital Episode Statistics; ICD-10, International Classification of Diseases, 10th Revision; PsA, psoriatic arthritis; SRP, self-reported psoriasis; SRPM, Self-reported psoriasis-relevant medication.

**Supplementary Table S2:** Full Results Using Selected Controls

Candidate Psoriasis Phenotype	Ncases	Ncontrols	minPPV, Mean (95% CI)	Power to Detect Common Weak Effect	Power to Detect Common Strong Effect	Power to Detect Rare Weak Effect	Power to Detect Rare Strong Effect
**Single data source**							
SRP	4,244	141,279	0.669 (0.612–0.726)	0.478	0.994	0.166	0.628
SRPM	1,927	141,279	0.739 (0.652–0.826)	0.296	0.922	0.124	0.410
HESmain	289	141,279	0.605 (0.422–0.788)	0.077	0.201	0.066	0.106
HESsec	1,532	141,279	0.587 (0.491–0.683)	0.175	0.674	0.083	0.236
HESany	1,726	141,279	0.579 (0.489–0.668)	0.178	0.716	0.089	0.265
GPraw	7,956	141,279	0.324 (0.279–0.370)	0.243	0.849	0.097	0.331
GP	5,768	141,279	0.464 (0.405–0.523)	0.340	0.953	0.126	0.451
GP2	2,422	141,279	0.586 (0.503–0.669)	0.242	0.860	0.107	0.337
GP3	1,389	141,279	0.614 (0.515–0.714)	0.172	0.675	0.087	0.239
**Combined data sources**							
1-SRP-HESany	5,194	141,279	0.624 (0.570–0.677)	0.499	0.996	0.165	0.643
1-SRP-GP	8,647	141,279	0.517 (0.471–0.563)	0.543	0.999	0.176	0.696
1-SRP-GP2	5,786	141,279	0.615 (0.559–0.670)	0.538	0.997	0.177	0.671
1-SRP-HESany-GP	9,316	141,279	0.508 (0.463–0.553)	0.561	0.999	0.182	0.709
1-SRP-HESany-GP2	6,574	141,279	0.585 (0.535–0.636)	0.542	0.999	0.185	0.689
2-SRP-HESany-GP	2,122	141,279	0.721 (0.638–0.805)	0.303	0.936	0.122	0.423
All-SRP-HESany-GP	300	141,279	0.818 (0.616–1.020)	0.105	0.326	0.064	0.136
2-SRP-SRM-HESany-GP	3,499	141,279	0.696 (0.628–0.763)	0.443	0.987	0.157	0.567
2-SRP-SRM-HESany-GP2	2,965	141,279	0.726 (0.660–0.792)	0.416	0.981	0.142	0.536
3-SRP-SRM-HESany-GP	1,122	141,279	0.771 (0.675–0.866)	0.216	0.776	0.090	0.289
All-SRP-SRM-HESany-GP	185	141,279	0.870 (0.637–1.104)	0.084	0.253	0.065	0.124
**Phenotypes incorporating PsA codings**							
SRP+PsA	4,603	141,279	0.664 (0.606–0.721)	0.503	0.995	0.163	0.650
SRPM+PsA	2,107	141,279	0.747 (0.661–0.832)	0.330	0.949	0.120	0.449
HESany+PsA	2,272	141,279	0.616 (0.530–0.703)	0.252	0.866	0.100	0.356
GP+PsA	6,024	141,279	0.457 (0.398–0.517)	0.348	0.957	0.123	0.450
1-SRP-HESany-GP+PsA	9,864	141,279	0.510 (0.464–0.555)	0.582	0.999	0.195	0.739
2-SRP-HESany-GP+PsA	2,579	141,279	0.713 (0.629–0.797)	0.348	0.969	0.131	0.487
2-SRP-SRM-HESany-GP+PsA	3,917	141,279	0.688 (0.620–0.756)	0.468	0.994	0.154	0.609

Abbreviations: CI, confidence interval; GP, general practitioner; HES, Hospital Episode Statistics; MAF, minor allele frequency; minPPV, lower bound of positive predictive value for psoriasis phenotype (i.e. inverse variance-weighted regression slope); OR, odds ratio; PsA, psoriatic arthritis; SRP, self-reported psoriasis; SRPM, self-reported psoriasis-relevant medication.

Candidate psoriasis phenotype abbreviations are defined in Table 1. The definition of scenarios used in power calculations is as follows – common risk factor, MAF: 30%; rare risk factor, MAF: 5%; weak effect, OR: 1.1; strong effect, OR: 1.25.

**Supplementary Table S3:** Full Results Using Unselected Controls

Candidate Psoriasis Phenotype	Ncases	Ncontrols	IVW Estimate, Mean (95% CI)	Power to Detect Common Weak Effect	Power to Detect Common Strong Effect	Power to Detect Rare Weak Effect	Power to Detect Rare Strong Effect
**Single data source**							
SRP	4,244	332,489	0.657 (0.602–0.712)	0.486	0.995	0.161	0.628
SRPM	1,927	334,806	0.723 (0.639–0.807)	0.306	0.922	0.116	0.412
HESmain	289	336,444	0.586 (0.403–0.769)	0.078	0.200	0.070	0.107
HESsec	1,532	335,201	0.570 (0.474–0.666)	0.174	0.685	0.086	0.243
HESany	1,726	335,007	0.562 (0.473–0.651)	0.182	0.715	0.090	0.260
GPraw	7,956	328,777	0.311 (0.268–0.355)	0.250	0.854	0.099	0.334
GP	5,768	330,965	0.451 (0.393–0.509)	0.339	0.958	0.119	0.471
GP2	2,422	334,311	0.570 (0.488–0.651)	0.253	0.856	0.108	0.346
GP3	1,389	335,344	0.596 (0.497–0.694)	0.181	0.673	0.086	0.240
**Combined data sources**							
1-SRP-HESany	5,194	331,539	0.613 (0.562–0.665)	0.515	0.997	0.166	0.651
1-SRP-GP	8,647	328,086	0.510 (0.466–0.554)	0.565	0.999	0.185	0.709
1-SRP-GP2	5,786	330,947	0.605 (0.551–0.658)	0.540	0.999	0.174	0.684
1-SRP-HESany-GP	9,316	327,417	0.502 (0.459–0.544)	0.574	0.999	0.190	0.728
1-SRP-HESany-GP2	6,574	330,159	0.577 (0.528–0.625)	0.545	0.999	0.175	0.699
2-SRP-HESany-GP	2,122	334,611	0.706 (0.623–0.789)	0.319	0.936	0.117	0.430
All-SRP-HESany-GP	300	336,433	0.798 (0.598–0.999)	0.092	0.327	0.063	0.132
2-SRP-SRM-HESany-GP	3,499	333,234	0.683 (0.617–0.748)	0.451	0.992	0.148	0.583
2-SRP-SRM-HESany-GP2	2,965	333,768	0.712 (0.648–0.776)	0.422	0.984	0.147	0.551
3-SRP-SRM-HESany-GP	1,122	335,611	0.753 (0.658–0.848)	0.212	0.773	0.095	0.295
All-SRP-SRM-HESany-GP	185	336,548	0.850 (0.617–1.084)	0.088	0.248	0.061	0.123
**Phenotypes incorporating PsA codings**							
SRP+PsA	4,603	332,130	0.652 (0.598–0.707)	0.516	0.997	0.174	0.655
SRPM+PsA	2,107	334,626	0.731 (0.649–0.814)	0.328	0.947	0.123	0.444
HESany+PsA	2,272	334,461	0.600 (0.516–0.685)	0.254	0.872	0.100	0.362
GP+PsA	6,024	330,709	0.445 (0.387–0.503)	0.351	0.960	0.125	0.464
1-SRP-HESany-GP+PsA	9,864	326,869	0.504 (0.461–0.548)	0.592	1.000	0.192	0.751
2-SRP-HESany-GP+PsA	2,579	334,154	0.698 (0.616–0.780)	0.366	0.967	0.131	0.480
2-SRP-SRM-HESany-GP+PsA	3,917	332,816	0.675 (0.610–0.741)	0.471	0.995	0.161	0.624

Abbreviations: CI, confidence interval; GP, general practitioner; HES, Hospital Episode Statistics; IVW, inverse variance-weighted; MAF, minor allele frequency; minPPV, lower bound of positive predictive value for psoriasis phenotype (i.e. IVW regression slope); OR, odds ratio; PsA, psoriatic arthritis; SRP, self-reported psoriasis; SRPM, self-reported psoriasis-relevant medication.

Candidate psoriasis phenotype abbreviations are defined in Table 1. Definition of scenarios used in power calculations as follows – common risk factor, MAF: 30%; rare risk factor, MAF: 5%; weak effect, OR: 1.1; strong effect, OR: 1.25.

**Supplementary Table S4:** IVW Estimates (and 95% CI) for Positive Control Dermatologist-Diagnosed Psoriasis Case-Control Cohorts

Cohort	Number of Psoriasis Cases	Number of Controls	IVW Mean (95% CI)
**Tsoi et al. cohorts**			
PsA	1,430	1,417	0.896 (0.774–1.018)
CASP	1,352	1,389	1.098 (0.967–1.230)
Kiel	464	1,135	1.212 (1.023–1.395)
Genizon	761	993	0.939 (0.778–1.101)
WTCCC2	2,178	5,175	1.074 (0.984–1.163)
Exomechip	3,863	4,028	0.927 (0.854–1.000)
PAGE Immunochip	3,181	7,406	0.966 (0.891–1.040)
**External cohort**			
BSTOP	3,864	6,900	1.063 (0.980–1.146)

Abbreviations: BSTOP, Biomarkers of Systemic Treatment Outcomes in Psoriasis; CASP, Collaborative Association Study of Psoriasis; CI, confidence interval; IVW, inverse variance-weighted; PsA, psoriatic arthritis; WTCCC2, Wellcome Trust Case Control Consortium 2.

**Supplementary Table S5:** Summary Statistics for 35 Psoriasis Loci Used as Genetic Instruments in Regressions

RSID	Coordinates (hg19)	RA	NRA	Effect Size	Standard Error	*P*-Value
rs10794648	chr1:24518206	C	T	0.1808	0.0211	1.02 × 10^−17^
rs113935720	chr1:67713346	T	C	0.3574	0.0381	6.08 × 10^−21^
rs4845453	chr1:152591953	C	G	0.1792	0.0181	3.70 × 10^−23^
rs35194171	chr2:61072183	A	T	0.1617	0.0173	9.20 × 10^−21^
rs13024541	chr2:62555737	T	C	0.1090	0.0173	2.93 × 10^−10^
rs17715343	chr2:163167746	C	G	0.3052	0.0334	7.10 × 10^−20^
rs13080782	chr3:16996623	G	A	0.1198	0.0175	7.82 × 10^−12^
rs11928138	chr3:101641048	C	T	0.1273	0.0223	1.07 × 10^−8^
rs27044	chr5:96118852	G	C	0.1499	0.0185	5.63 × 10^−16^
rs847	chr5:131996669	C	T	0.1789	0.0217	1.48 × 10^−16^
rs74817271	chr5:150469973	A	G	0.4808	0.0326	2.73 × 10^−49^
rs12188300	chr5:158829527	T	A	0.5075	0.0288	1.82 × 10^−69^
rs9504361	chr6:577820	A	G	0.0958	0.0174	3.72 × 10^−8^
rs7748720	chr6:20689945	A	G	0.1583	0.0208	2.67 × 10^−14^
rs9481169	chr6:111929862	T	G	0.3660	0.0280	3.52 × 10^−39^
rs582757	chr6:138197824	C	T	0.1791	0.0186	6.55 × 10^−22^
rs2485363	chr6:159506121	T	G	0.0993	0.0179	2.70 × 10^−8^
rs11767350	chr7:37385365	A	G	0.1018	0.0175	5.55 × 10^−9^
rs11795343	chr9:32523737	T	C	0.1102	0.0173	2.04 × 10^−10^
rs11531804	chr9:110792282	T	C	0.1040	0.0183	1.43 × 10^−8^
rs2459446	chr10:75601596	C	T	0.1145	0.0177	1.05 × 10^−10^
rs1108618	chr10:81043743	A	G	0.1105	0.0176	3.56 × 10^−10^
rs600377	chr11:64124940	T	G	0.1050	0.0178	3.74 × 10^−9^
rs7936694	chr11:109968706	A	G	0.1378	0.0176	5.27 × 10^−15^
rs3802826	chr11:128406438	A	G	0.1140	0.0171	2.32 × 10^−11^
12:56738816:D	chr12:56738816	GC	G	0.3517	0.0367	8.89 × 10^−22^
rs12883343	chr14:35852474	G	C	0.1544	0.0175	9.92 × 10^−19^
rs28624578	chr15:31637666	T	C	0.1626	0.0282	8.26 × 10^−9^
rs12447930	chr16:31023273	A	G	0.1309	0.0177	1.42 × 10^−13^
rs28998802	chr17:26124908	A	G	0.2189	0.0238	3.91 × 10^−20^
rs8070763	chr17:40536396	C	T	0.1064	0.0179	3.01 × 10^−9^
rs3785437	chr17:73906222	T	G	0.1397	0.0254	3.97 × 10^−8^
rs34536443	chr19:10463118	G	C	0.6846	0.0553	3.49 × 10^−35^
rs492602	chr19:49206417	G	A	0.1059	0.0170	4.24 × 10^−10^
rs5754387	chr22:21974703	C	G	0.1296	0.0213	1.16 × 10^−9^

Abbreviations: RA, psoriasis risk allele; NRA, psoriasis nonrisk allele.

## Supplementary Materials and Methods

### UK Biobank data and phenotype definition

This project used UK Biobank data under approved project number 15147. UK Biobank is a prospective study with >500,000 participants aged 40–69 years when recruited in 2006–2010 (Bycroft et al., 2018). The study has collected and continues to collect extensive phenotypic and genotypic detail about its participants, including data from questionnaires, physical measures, sample assays, accelerometry, multimodal imaging, genome-wide genotyping, and longitudinal follow-up for a wide range of health-related outcomes. Linkage to health record data comprises Hospital Episode Statistics data that document hospital inpatient visits and primary care data (currently available for 230,105 participants). The UK Biobank study was approved by the National Health Service National Research Ethics Service (approval nos. 11/NW/0382, 16/NW/0274), and all participants provided written informed consent.

We defined nine candidate psoriasis definitions based on a single-source data type (Table 1): two based on self-reported illnesses and medications, three based on linked Hospital Episode Statistics data, and four based on linked primary care data. Full details of the codes included are given in Supplementary Table S1. Self-reported medications include prescription medications being taken regularly by participants at the time of their assessment center visit (UK Biobank field 20003). The full list of medications reported by participants self-reporting psoriasis was reviewed by dermatologists (SKM, CHS) to identify relevant psoriasis medications. Linked primary care data include two types of read code: readV2 and readCTV3 (NHS Digital, 2020). We included codes of both types. We further distinguished candidate primary care phenotypes based on read codes that corresponded to International Classification of Diseases, 10th Revision, L40 codes in a UK Biobank mapping file (Table 1: GPraw) (UK Biobank, 2021) from those based on a previously validated list of psoriasis read codes (Table 1: GP) (Seminara et al., 2011). Validated read code lists in readV2 format were mapped to readCTV3 using the UK Biobank mapping file (UK Biobank, 2021).

We further considered candidate psoriasis definitions based on combining data sources. These ranged from broader definitions in which a single psoriasis coding across data sources would be sufficient, to stricter definitions requiring psoriasis codings from multiple data sources (Table 1). Because psoriatic arthritis typically presents with skin lesions, we considered additional candidate psoriasis definitions based on expanded lists of self-report, primary care (Ogdie et al., 2013), and Hospital Episode Statistics codes that included psoriatic arthritis (Table 1; Supplementary Table S1).

### UK Biobank genotype data and association testing

The UK Biobank central team performed genotype calling and imputation. Genotyping was performed using the Affymetrix UK BiLEVE Axiom array (n ∼50,000) and the Affymetrix UK Biobank Axiom array (n ∼450,000) (Bycroft et al., 2018). Based on quality control metrics provided by UK Biobank, we removed samples that exhibited gender mismatch, excess relatedness, heterozygosity, or missingness > 5% and extracted individuals determined by UK Biobank to form an unrelated subset of homogeneous (White British) ancestry. We then removed additional individuals with low call rates (<98%) in well-called (>90%) markers, giving 336,814 samples for subsequent analysis (336,733 after withdrawals). Genome-wide imputation was performed by the UK Biobank central team using IMPUTE2 software and a reference panel derived from UK10K and 1,000 Genomes phase 3 haplotypes (Howie et al., 2011, 2009). For subsequent analysis, we considered variants with imputation *R*^2^ > 0.7 and minor allele frequency > 0.5%.

We performed association testing at 35 variants of interest (see in subsequent section) for each candidate psoriasis definition we generated. Each definition provided a set of participants to be considered for psoriasis cases. For unaffected controls, we included participants who had linked primary care data and were negative for psoriasis under all candidate psoriasis definitions (n = 141,279). We fitted a logistic regression for each variant using PLINK v2.0 (Chang et al., 2015), using 20 ancestry principal components and genotyping array as covariates.

### Genetic instrument selection and regression

To derive a reference genetic instrument representative of dermatologist-diagnosed psoriasis, summary statistics from seven dermatologist-derived case-control GWASs (totaling 13,229 cases and 21,543 controls) (Tsoi et al., 2017) were analyzed using an inverse variance–weighted (IVW) fixed effect meta-analysis. We identified 38 independent genome-wide significant (*P* < 5 × 10^−8^) associations at least 1 Mb apart. This excluded associations in the major histocompatibility complex region on chromosome 6: the strong association between *HLA-C**∗**06:02* and psoriasis age of onset means that estimated effect sizes at this locus are strongly influenced by ascertainment strategy, and comparison across studies is complex. Of the 38 lead variants, 35 were available in the UK Biobank imputed genetic dataset, whereas the remaining 3 were unavailable with no suitable proxy found using the LDLink platform (Machiela and Chanock, 2015).

For each candidate UK Biobank psoriasis definition, effect sizes (betas) at the 35 lead variants were regressed against effect sizes from the reference instrument (Supplementary Table S5), weighted by inverse variance to give higher weight to loci with more confident effect size estimates, using function *mr_ivw* from R package MendelianRandomization (version 0.5.0) (Yavorska and Burgess, 2017). The slope of the regression line gives an indication of how depressed, on average, are the effect sizes of the candidate psoriasis phenotype in comparison to established psoriasis effect sizes (Figure 1); a slope of 1 would indicate effect sizes consistent with those already established for dermatologist-derived psoriasis (full results in Supplementary Table S2). To understand how accuracy and statistical power would be affected by a less stringent definition of controls, we fitted alternative regression models in which all participants not positive for the candidate psoriasis definition were included as “unselected” controls (Supplementary Table S3). We observed slightly higher regression slopes when using selected controls, in comparison to using unselected controls (Supplementary Figure S4).

### IVW regression slope interpretation

With the assumption that the unaffected control group of UK Biobank participants were representative of the control datasets in dermatologist-derived psoriasis GWAS, we considered that any effect size depression relative to the reference genetic instrument could be driven by the inclusion of misclassified individuals within the psoriasis cases (compared with the specialist-diagnosed psoriasis cohorts from the study by Tsoi et al.). This may be through incorrect self-report or misdiagnosis by nondermatologists in linked Hospital Episode Statistics or primary care data. At any single locus, the degree of effect size depression will depend on the positive predictive value (PPV; true positives / true positives + false positives) of the candidate UK Biobank definition but also on the allele frequency and the magnitude of the established effect. The relationship between the PPV and the IVW regression slope is therefore complex, and we undertook simulations to inform our interpretation of IVW regression slopes. Using PLINK v1.9 (Chang et al., 2015), we simulated genetic datasets representing affected and unaffected individuals for 35 SNVs with effect size and frequency equivalent to those in the reference genetic instrument. In each simulation, we included 125,000 controls from simulated unaffected individuals, and 5,000 cases that were a mix of simulated affected (true positive) and unaffected (false positive) individuals. The simulated PPV was varied from 0% to 100% in 10% increments, and we performed 1,000 simulations per dilution level. We calculated the IVW slope for each simulation and observed an approximately linear relationship between the IVW slope and PPV (Supplementary Figure S1). In UK Biobank, effect size depression relative to the reference genetic instrument may not be exclusively because of false positives (individuals with incorrect psoriasis diagnoses/self-reports), but also to individuals with milder psoriasis (with lower genetic liability than typical specialist-ascertained cases). Our IVW slopes can therefore be interpreted as representing the lower bound of the PPV for each candidate UK Biobank psoriasis phenotype. To ensure that effect sizes were not systematically affected by difference in risk allele frequencies between the population in the study by Tsoi et al. and UK Biobank populations (owing to differences in ancestry), risk allele frequencies were compared between the control populations of the two studies (Supplementary Figure S5) and shown to be highly concordant. 23andMe data were excluded from the Tsoi et al. cohorts, and the Collaborative Association Study of Psoriasis cohort genotype data were unavailable.

### Power calculations

For each candidate UK Biobank psoriasis phenotype, we estimated the statistical power to detect association with a novel genetic or epidemiological psoriasis risk factor. We took an empirical approach, based on a simple 2 × 2 contingency table that assumed true population proportions P_EA_ (exposed, affected), P_NA_ (not exposed, affected), P_EU_ (exposed, unaffected), and P_NU_ (not exposed, unaffected). For a specified odds ratio (P_EA_P_NU_/P_NA_P_EU_), psoriasis prevalence (P_A_ = P_EA_ + P_NA_), and risk factor prevalence (P_E_ = P_EA_ + P_EU_), implied values for these proportions can be calculated using the fact that proportions sum to 1. For each candidate UK Biobank psoriasis definition, these population proportions were used to simulate case and control samples of the same size, with the IVW slope estimate determining the simulated PPV. A Bernoulli function was used to generate risk factor–exposed and unexposed individuals, using P_EA_/P_A_ as the probability of true positive cases being exposed to the risk factor, and P_EU_/P_U_ as the probability of false positive cases and controls being exposed to the risk factor. Each power calculation was based on 10,000 simulations, with power estimated as the proportion of simulations for which a 2 × 2 chi-squared statistic was above 3.841 (*P* < 0.05).

For all power calculations, we assumed a psoriasis prevalence of 2%. For each candidate psoriasis definition, we calculated power for four scenarios based on a hypothetical risk factor’s population frequency (common: 30%; rare: 5%) and odds ratio (weak: 1.1; strong: 1.25). For brevity, only the power estimates for common, weak risk factors are presented in the main text. When estimating power for discovery using unselected controls, we took the estimate of true case dilution (PPV) from the IVW slope corresponding to selected controls. This is because the case group is not influenced by control selection, and our simulations that demonstrated a linear relationship were based on selected controls.
